# Immunogenetics of longevity and its association with human endogenous retrovirus K

**DOI:** 10.3389/fragi.2025.1471202

**Published:** 2025-02-04

**Authors:** Lisa M. James, Apostolos P. Georgopoulos

**Affiliations:** ^1^ Department of Veterans Affairs Health Care System, The HLA Research Group, Brain Sciences Center, Minneapolis, MN, United States; ^2^ Department of Neuroscience, University of Minnesota Medical School, Minneapolis, MN, United States; ^3^ Department of Psychiatry, University of Minnesota Medical School, Minneapolis, MN, United States; ^4^ Institute for Health Informatics, University of Minnesota Medical School, Minneapolis, MN, United States

**Keywords:** longevity, human leukocyte antigen (HLA), viruses, human endogenous retrovirus K (HERV-K), apolipoprotein E

## Abstract

**Introduction:**

The human immune system is equipped to neutralize and eliminate viruses and other foreign antigens via binding of human leukocyte antigen (HLA) molecules with foreign antigen epitopes and presenting them to T cells. HLA is highly polymorphic, resulting in subtle differences in the binding groove that influence foreign antigen binding and elimination. Here we tested the hypothesis that certain HLA alleles may promote longevity by enhanced ability to counter virus antigens that may otherwise contribute to morbidity and mortality.

**Methods:**

We utilized high-resolution genotyping to characterize HLA and apolipoprotein E in a large sample (N = 986) of participants (469 men, 517 women) ranging in age from 24 to 90+ years old (mean age: 58.10 years) and identified 244 HLA alleles that occurred in the sample. Since each individual carries 12 classical HLA alleles (6 alleles of each Class I and Class II), we determined *in silico* the median predicted binding affinity for each individual (across the 12 HLA alleles) and each of 13 common viruses (Human Herpes Virus 1 [HHV1], HHV2, HHV3, HHV4, HHV5, HHV6A, HHV6B, HHV7, HHV8, human papilloma virus [HPV], human polyoma virus [JCV], human endogenous retrovirus K [HERVK], and HERVW). Next, we performed a stepwise multiple linear regression where the age of the participant was the dependent variable and the 13 median predicted HLA-virus binding affinities were the independent variables.

**Results:**

The analyses yielded only one statistically significant effect–namely, a positive association between age and HERVK (P = 0.005). Furthermore, we identified 13 HLA alleles (9 HLA-I and 4 HLA-II) that occurred at greater frequency in very old individuals (age ≥90 years) as compared to younger individuals. Remarkably, for those 13 alleles, the predicted binding affinities were significantly higher for HERVK than for the other viruses (P < 0.001). ApoE genotypes did not differ significantly between older and younger groups.

**Discussion:**

Taken together, the results showed that HLA-HERVK binding affinity is a robust predictor of longevity and that HLA alleles that bind with high affinity to HERVK were enriched in very old individuals. The findings of the present study highlight the influence of interactions between host immunogenetics and virus exposure on longevity and suggest that specific HLA alleles may promote longevity via enhanced immune response to specific common viruses, notably HERVK.

## 1 Introduction

Exposure to viruses and other foreign antigens is associated with numerous and wide-ranging health conditions and sequelae (Chang et al., 2017; Mui et al., 2017; Krump and You, 2018; Houen and Trier, 2021; Hussein and Rahal, 2019; Lotz et al., 2021; Al-Haddad et al., 2019; Burgdorf et al., 2019). Under optimal conditions–that is, immunocompetence–the human immune system is equipped to neutralize and eliminate foreign antigens that may otherwise contribute to morbidity and mortality. Specifically, the Human Leukocyte Antigen (HLA) region of chromosome 6 codes for cell-surface glycoproteins that play a critical role in the host immune response to foreign antigens including viruses, bacteria, and cancer neoantigens. Each individual possesses 12 classical HLA alleles, inherited in a Mendelian fashion, including six each from Class I (HLA-A, B, C) and Class II (HLA-DPB1, DQB1, DRB1). The steps involved in antigen processing and presentation by HLA-I and HLA-II alleles are reviewed elsewhere (Pishesha et al., 2022). Briefly, HLA-I molecules, which are expressed on all nucleated cells, help clear foreign antigens via binding and transporting cytosolic foreign antigen epitopes to the cell surface for presentation to cytotoxic CD8^+^ T cells to signal destruction of an infected cell. HLA-II molecules, which are expressed on lymphocytes and professional antigen presenting cells, bind and present endocytosed exogenous antigen epitopes to CD4^+^ T cells to stimulate antibody production and adaptive immunity. The HLA region is the most highly polymorphic of the human genome (Trowsdale and Knight, 2013). Even single amino acid differences can alter HLA-antigen binding (Hov et al., 2011), thereby influencing foreign antigen elimination and disease susceptibility (Dendrou et al., 2018). A recent genome-wide association meta-analysis documented that the HLA-DRB1 region is also associated with longevity as is apolipoprotein E (apoE) (Joshi et al., 2017). Here we tested the hypothesis that certain HLA alleles may promote longevity by enhanced ability to eliminate virus antigens that may otherwise contribute to morbidity and mortality. Specifically, we utilized high-resolution genotyping to 1) characterize the composition of HLA in a large sample of participants, 2) evaluate *in silico* the binding affinity of their HLA alleles with common viruses, and 3) determine the association between HLA-virus antigen binding affinity and age. We also evaluated the influence of apoE on longevity.

## 2 Materials and methods

### 2.1 Participants

A total of 986 participants (469 men, 517 women), the majority (87%) of whom were United States (US) veterans, were included in the present analyses. Participants were excluded from participation if they had been diagnosed with medical or psychiatric conditions that could impair ability to provide informed consent or participate in the study (e.g., Alzheimer’s dementia, schizophrenia). All participants provided written informed consent and were compensated for their participation. The study was approved by the Minneapolis Veterans Affairs Healthcare System Institutional Review Board and all research was performed in accordance with relevant guidelines and regulations.

### 2.2 HLA genotyping

DNA isolation was carried out from whole blood or saliva samples using commercially available kits (blood: ArchivePure cat. 2300730 from 5Prime distributed by Fisher Scientific or VWR; saliva: Oragene-Discover cat. OGR-500 coupled with prepIT purifier reagent cat. PT-L2P/DNA Genotek Inc. Ottawa, ON, Canada). The purified DNA samples were sent to HistoGenetics (http://www.histogenetics.com/) for high-resolution HLA Sequence-based Typing (SBT; details are given in https://bioinformatics.bethematchclinical.org/HLA-Resources/HLA-Typing/High-Resolution-Typing-Procedures/ and https://bioinformatics.bethematchclinical.org/WorkArea/DownloadAsset.aspx?id=6482). Their sequencing DNA templates are produced by locus- and group-specific amplifications that include exon 2 and 3 for Class I (A, B, C) and exon 2 for Class II (DRB1, DRB3/4/5, DQB1, and DPB1) and reported as Antigen Recognition Site (ARS) alleles as per ASHI recommendation (Cano et al., 2007).

### 2.3 ApoE genotyping

DNA samples were genotyped using PCR amplification followed by restriction enzyme digestion (Reymer et al., 1995). Each amplification reaction contained PCR buffer with 15 mmol/L MgCl_2_ ng amounts of genomic DNA, 20 pmol apoE forward (5N TAA GCT TGG CAC GGC TGT CCA AGG A 3N) and reverse (5N ATA AAT ATA AAA TAT AAA TAA CAG AAT TCG CCC CGG CCT GGT ACA C 3N) primers, 1.25 mmol/L of each deoxynucleotide triphosphate, 10% dimethylsulfoxide, and 0.25 μL AmpliTaq DNA polymerase. Reaction conditions in a thermocycler included an initial denaturing period of 3 min at 95 C, 1 min at 60 C, and 2 min at 72 C; followed by 32 cycles of 1 min at 95 C, 1 min at 60 C, and 2 min at 72 C; and a final extension of 1 min at 95 C, 1 min at 60 C, and 3 min at 72 C. PCR products were digested with *HhaI* and separated on a 4% Agarose gel which was stained with ethidium bromide. Known apoE isoform standards were included in the analysis.

### 2.4 Viruses

Thirteen viruses implicated in various diseases in humans were investigated, including 9 human herpes viruses (HHV1, HHV2, HHV3, HHV4, HHV5, HHV6A, HHV6B, HHV7, HHV8), human polyoma JC virus (JCV), human papilloma virus 16 (HPV), human endogenous retrovirus K (HERVK), and human endogenous retrovirus W (HERVW). Details of the viral proteins used are given in Table 1.

**TABLE 1:** Viral proteins used. HHV, human herpes virus; JCV, human polyomavirus JC; HPV, human papillomavirus; HERVK, human endogenous retrovirus K; HERVW, human endogenous retrovirus W.

Index	Virus	Protein description	UniprotKB ID
1	HHV1	Envelope glycoprotein D	Q69091
2	HHV2	Envelope glycoprotein D	P03172
3	HHV3	Envelope glycoprotein E	Q9J3M8
4	HHV4	Envelope glycoprotein B	P03188
5	HHV5	Envelope glycoprotein B	P06473
6	HHV6A	Envelope Glycoprotein Q2	P0DOE0
7	HHV6B	Envelope Glycoprotein Q1	Q9QJ11
8	HHV7	Envelope glycoprotein H	P52353
9	HHV8	Envelope glycoprotein H	F5HAK9
10	JCV	Major capsid protein VP1	P03089
11	HPV	Major capsid protein L1	Q81007
12	HERVK	10 Pol protein	P10266
13	HERVW	Envelope protein	Q9UQF0

### 2.5 In silico determination of predicted binding affinity of HLA-I and HLA-II alleles

**FIGURE 1:**
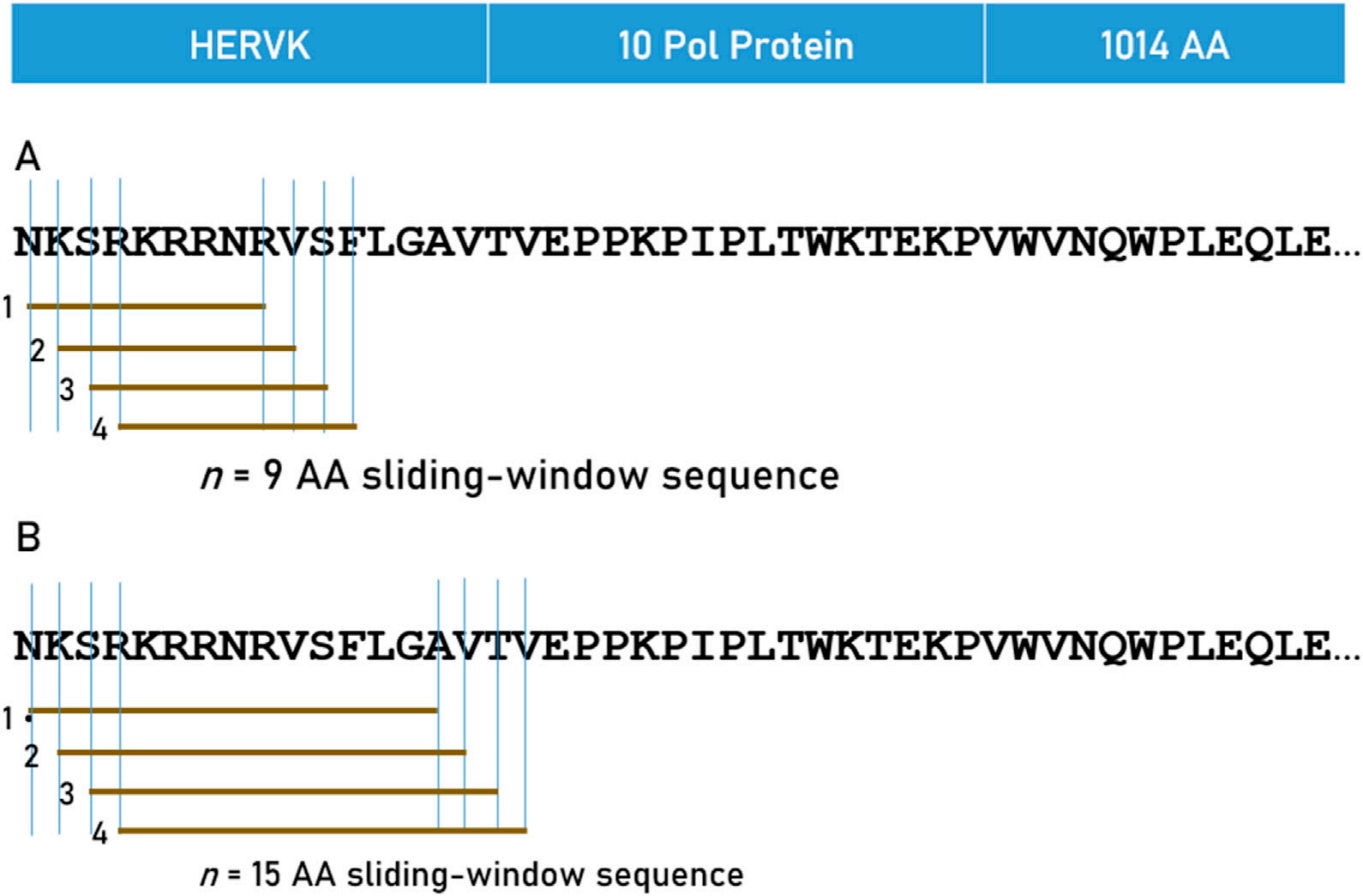
The 9-AA sliding window approach used for HLA Class I **(A)** and 15-AA window for HLA Class II **(B)** analyses are illustrated for HERVK.

Predicted binding affinities were obtained for viral protein epitopes using the Immune Epitope Database (IEDB) NetMHCpan (ver. 4.1) tool (Reynisson et al., 2020; IEDB, 2020). More specifically, we used the sliding window approach (Charonis et al., 2020a; Charonis et al., 2020b; Charonis et al., 2021) to test exhaustively all possible linear 9-mer (for HLA-I predictions) and 15-mer (for HLA-II predictions) AA residue epitopes of the 13 viral proteins analyzed (Table 1; Supplementary Table S1). The method is illustrated in Figure 1 for the HERVK virus protein. For each epitope-HLA molecule tested, this tool gives, as an output, the percentile rank of binding affinity of the HLA molecule and the epitope, among predicted binding affinities of the same HLA molecule to a large number of different peptides of the same AA length; the smaller the percentile rank, the better the binding affinity. Now, given a protein of *N* amino acid length and an epitope length of *k* AA, there are *N-k* binding affinity predictions, i.e., *N-k* percentile ranks. Of these predictions, for each viral protein and HLA molecule tested, we retained the lowest percentile rank (LPR) as the best binding affinity of the protein-HLA molecule pair. Finally, we took the inverse of LPR, so that higher values mean better binding affinities for more intuitive interpretation Equation 1:

$$\text{Predicted Binding Affinity }\left(\text{PBA}\right)=1/\text{LPR}$$ (1)

### 2.6 Statistical analyses

Standard statistical methods were used to analyze the data using the IBM-SPSS statistical package (version 29). For multidimensional scaling (MDS), the ALSCAL procedure of the IBM-SPSS package was used (Level, ordinal; Condition, Matrix; Model, Euclid; Dimension [2,2]; Criteria: S-stress convergence = 0.001, minimum s-stress = 0.005, number of iterations = 30), and the K-means clustering procedure of the same package for identifying clusters in the MDS plot (Number of clusters = 3, Method: Iterate and Classify).

## 3 Results

### 3.1 Age

**FIGURE 2:**
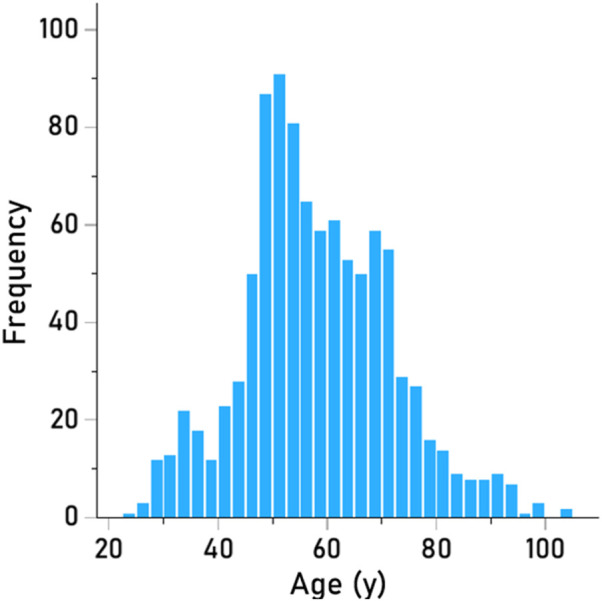
Frequency distribution of ages (N = 986).

The frequency distribution of age is shown in Figure 2; mean = 58.10 y, SD = 13.77 y, median = 56.67 y, minimum = 24.08 y, maximum = 90 + y.

### 3.2 HLA alleles

There were 244 distinct HLA alleles (142 of Class I and 93 of Class II) comprising the classical genes of Class I (A, B, C) and Class II (DPB1, DQB1, DRB1). The alleles and their frequencies (percentages) in our sample (N = 986) are given in Tables 2, 3.

**TABLE 2:** Alleles of HLA Class I, Genes A, B, C found in our sample (N = 986 participants).

	HLA-I gene A			HLA-I gene B				HLA-I gene C			
Index	Allele	N	Frequency (%)	Index	Allele	N	Frequency (%)	Index	Allele	N	Frequency (%)
1	A*01:01	290	29.412	1	B*07:02	292	29.615	1	C*01:02	74	7.505
2	A*02:01	541	54.868	2	B*07:05	8	0.811	2	C*02:01	1	0.101
3	A*02:02	4	0.406	3	B*07:04	1	0.101	3	C*02:02	115	11.663
4	A*02:05	18	1.826	4	B*08:01	221	22.414	4	C*02:10	1	0.101
5	A*02:06	4	0.406	5	B*13:02	46	4.665	5	C*03:02	8	0.811
6	A*02:17	1	0.101	6	B*14:01	23	2.333	6	C*03:03	99	10.041
7	A*02:30	1	0.101	7	B*14:02	61	6.187	7	C*03:04	191	19.371
8	A*02:35	1	0.101	8	B*14:03	1	0.101	8	C*04:01	192	19.473
9	A*02:63	1	0.101	9	B*15:01	140	14.199	9	C*04:09	1	0.101
10	A*02:77	2	0.203	10	B*15:03	3	0.304	10	C*05:01	140	14.199
11	A*03:01	308	31.237	11	B*15:07	1	0.101	11	C*05:07	1	0.101
12	A*03:02	2	0.203	12	B*15:09	2	0.203	12	C*06:02	159	16.126
13	A*03:81	1	0.101	13	B*15:10	1	0.101	13	C*07:01	301	30.527
14	A*11:01	113	11.46	14	B*15:16	2	0.203	14	C*07:02	311	31.542
15	A*11:02	1	0.101	15	B*15:17	3	0.304	15	C*07:04	30	3.043
16	A*23:01	40	4.057	16	B*15:18	2	0.203	16	C*07:19	1	0.101
17	A*24:02	169	17.14	17	B*15:24	2	0.203	17	C*08:01	2	0.203
18	A*24:03	4	0.406	18	B*15:35	1	0.101	18	C*08:02	84	8.519
19	A*25:01	45	4.564	19	B*18:01	78	7.911	19	C*08:03	1	0.101
20	A*26:01	46	4.665	20	B*18:09	1	0.101	20	C*12:02	12	1.217
21	A*26:08	4	0.406	21	B*27:02	13	1.318	21	C*12:03	84	8.519
22	A*26:12	1	0.101	22	B*27:04	1	0.101	22	C*14:02	23	2.333
23	A*29:01	6	0.609	23	B*27:05	89	9.026	23	C*15:02	41	4.158
24	A*29:02	49	4.97	24	B*27:07	2	0.203	24	C*15:04	1	0.101
25	A*30:01	29	2.941	25	B*27:08	1	0.101	25	C*15:05	8	0.811
26	A*30:02	13	1.318	26	B*27:10	1	0.101	26	C*15:06	1	0.101
27	A*30:04	2	0.203	27	B*35:01	97	9.838	27	C*15:09	1	0.101
28	A*31:01	54	5.477	28	B*35:02	11	1.116	28	C*16:01	70	7.099
29	A*32:01	74	7.505	29	B*35:03	30	3.043	29	C*16:02	3	0.304
30	A*33:01	15	1.521	30	B*35:08	10	1.014	30	C*16:04	2	0.203
31	A*33:03	11	1.116	31	B*35:17	1	0.101	31	C*16:50	1	0.101
32	A*34:01	1	0.101	32	B*37:01	24	2.434	32	C*17:01	11	1.116
33	A*34:02	2	0.203	33	B*38:01	29	2.941	33	C*18:01	2	0.203
34	A*36:01	3	0.304	34	B*39:01	30	3.043				
35	A*66:01	5	0.507	35	B*39:02	1	0.101				
36	A*68:01	81	8.215	36	B*39:05	2	0.203				
37	A*68:02	20	2.028	37	B*39:06	13	1.318				
38	A*68:37	1	0.101	38	B*39:24	1	0.101				
39	A*74:01	3	0.304	39	B*40:01	123	12.475				
40	A*74:03	1	0.101	40	B*40:02	31	3.144				
41	A*80:01	5	0.507	41	B*41:01	4	0.406				
				42	B*41:02	6	0.609				
				43	B*42:02	1	0.101				
				44	B*44:02	152	15.416				
				45	B*44:03	79	8.012				
				46	B*44:04	3	0.304				
				47	B*44:05	7	0.71				
				48	B*44:07	1	0.101				
				49	B*44:27	4	0.406				
				50	B*45:01	22	2.231				
				51	B*47:01	8	0.811				
				52	B*48:01	2	0.203				
				53	B*48:07	1	0.101				
				54	B*49:01	23	2.333				
				55	B*50:01	17	1.724				
				56	B*50:02	1	0.101				
				57	B*51:01	96	9.736				
				58	B*51:02	1	0.101				
				59	B*51:07	1	0.101				
				60	B*51:09	1	0.101				
				61	B*52:01	16	1.623				
				62	B*53:01	15	1.521				
				63	B*54:01	1	0.101				
				64	B*55:01	22	2.231				
				65	B*56:01	20	2.028				
				66	B*57:01	47	4.767				
				67	B*57:02	2	0.203				
				68	B*57:03	2	0.203				
				69	B*58:01	14	1.42				
				70	B*58:02	2	0.203				
				71	B*59:01	1	0.101				
				72	B*81:01	1	0.101				

**TABLE 3:** Alleles of HLA Class II, Genes DPB1, DQB1, DRB1 found in our sample (N = 986 participants).

HLA-II gene DPB1				HLA-II gene DQB1				HLA-II gene DRB1			
Allele		N	%	Allele		N	%	Allele		N	%
1	DPB1*01:01	100	10.142	1	DQB1*02:01	277	28.093	1	DRB1*01:01	161	16.329
2	DPB1*02:01	244	24.746	2	DQB1*02:02	137	13.895	2	DRB1*01:02	17	1.724
3	DPB1*02:02	7	0.71	3	DQB1*02:10	2	0.203	3	DRB1*01:03	12	1.217
4	DPB1*03:01	217	22.008	4	DQB1*03:01	358	36.308	4	DRB1*03:01	239	24.239
5	DPB1*04:01	811	82.252	5	DQB1*03:02	198	20.081	5	DRB1*03:02	2	0.203
6	DPB1*04:02	245	24.848	6	DQB1*03:03	74	7.505	6	DRB1*04:01	165	16.734
7	DPB1*05:01	48	4.868	7	DQB1*03:04	3	0.304	7	DRB1*04:02	6	0.609
8	DPB1*06:01	25	2.535	8	DQB1*03:05	1	0.101	8	DRB1*04:03	13	1.318
9	DPB1*09:01	15	1.521	9	DQB1*03:12	1	0.101	9	DRB1*04:04	78	7.911
10	DPB1*105:01	8	0.811	10	DQB1*03:19	10	1.014	10	DRB1*04:05	12	1.217
11	DPB1*10:01	32	3.245	11	DQB1*04:02	57	5.781	11	DRB1*04:06	3	0.304
12	DPB1*11:01	36	3.651	12	DQB1*05:01	214	21.704	12	DRB1*04:07	26	2.637
13	DPB1*124:01	3	0.304	13	DQB1*05:02	47	4.767	13	DRB1*04:08	11	1.116
14	DPB1*126:01	1	0.101	14	DQB1*05:03	47	4.767	14	DRB1*04:11	2	0.203
15	DPB1*131:00	1	0.101	15	DQB1*06:01	13	1.318	15	DRB1*07:01	229	23.225
16	DPB1*131:01	2	0.203	16	DQB1*06:02	279	28.296	16	DRB1*08:01	46	4.665
17	DPB1*13:01	30	3.043	17	DQB1*06:03	165	16.734	17	DRB1*08:02	3	0.304
18	DPB1*14:01	26	2.637	18	DQB1*06:04	61	6.187	18	DRB1*08:03	4	0.406
19	DPB1*15:01	12	1.217	19	DQB1*06:09	27	2.738	19	DRB1*08:04	6	0.609
20	DPB1*16:01	15	1.521	20	DQB1*06:84	1	0.101	20	DRB1*08:11	2	0.203
*21*	DPB1*17:01	*32*	*3.245*					21	DRB1*09:01	20	2.028
*22*	DPB1*18:01	*4*	*0.406*					22	DRB1*10:01	19	1.927
*23*	DPB1*19:01	*22*	*2.231*					23	DRB1*11:01	125	12.677
*24*	DPB1*20:01	*14*	*1.42*					24	DRB1*11:02	8	0.811
25	DPB1*23:01	13	1.318					25	DRB1*11:03	16	1.623
26	DPB1*29:01	1	0.101					26	DRB1*11:04	47	4.767
27	DPB1*350:01	1	0.101					27	DRB1*12:01	31	3.144
28	DPB1*35:01	1	0.101					28	DRB1*13:01	160	16.227
29	DPB1*40:01	1	0.101					29	DRB1*13:02	92	9.331
30	DPB1*46:01	1	0.101					30	DRB1*13:03	21	2.13
31	DPB1*50:01	1	0.101					31	DRB1*13:04	1	0.101
32	DPB1*57:01	1	0.101					32	DRB1*13:05	2	0.203
33	DPB1*59:01	1	0.101					33	DRB1*13:06	1	0.101
34	DPB1*92:01	1	0.101					34	DRB1*13:12	1	0.101
								35	DRB1*14:01	6	0.609
								36	DRB1*14:02	2	0.203
								37	DRB1*14:04	1	0.101
								38	DRB1*14:06	1	0.101
								39	DRB1*14:54	41	4.158
								40	DRB1*15:01	274	27.789
								41	DRB1*15:02	11	1.116
								42	DRB1*15:03	9	0.913
								43	DRB1*16:01	36	3.651
								44	DRB1*16:02	10	1.014

We used the age of 90 years as a conservative cut-off point to separate participants in 2 groups: Group1 (age <90 years, N = 964) and Group 2 (age ≥90 years, N = 22, “very old”). We hypothesized that surviving to very old age could/would be associated with the presence of specific HLA alleles. For that purpose, we searched for alleles with higher frequencies in Group 2 (as compared to Group 1) and found 13 alleles with significantly higher proportions in Group 2 than in Group 1 (Very Old Alleles, VOA); since we were testing the hypothesis of only higher allele frequencies in Group 2, we used a one-tailed test of proportions to obtain the statistical significance of the difference between the two proportions. These alleles with detailed statistics are shown in Table 4; they comprise 9 alleles of HLA Class I (3 of each A, B, and C genes) and 4 of Class II (1 of DPB1 gene, 1 of DQB1 gene, and 2 of DRB1 gene). The ratios of Group 2/Group 1 proportions ranged from 1.62 (allele A*03:01) to 6.43 (alleles C*03:02 and DRB1*11:02).

**TABLE 4:** HLA alleles occurring more frequently in the very old (Group 2).

		Group 1 (N = 964)		Group 2 (N = 22)		Comparison of proportions	
		<90 years		≥90 years			
Index	Allele	Counts	Proportion group 1	Counts	Proportion group 2	G2-G1	P (1-tailed; uncorrected)
1	A*02:05	16	0.017	2	0.091	0.074	0.005
2	A*03:01	297	0.308	11	0.500	0.192	0.027
3	A*29:02	46	0.048	3	0.136	0.089	0.029
4	B*27:05	81	0.084	8	0.364	0.280	3.01E-06
5	B*39:01	27	0.028	3	0.136	0.108	0.002
6	B*56:01	18	0.019	2	0.091	0.072	0.009
7	C*01:02	70	0.073	4	0.182	0.109	0.027
8	C*02:02	109	0.113	6	0.273	0.160	0.011
9	C*03:02	7	0.007	1	0.045	0.038	0.024
10	DQB1*05:01	204	0.212	10	0.455	0.243	0.003
11	DRB1*01:01	153	0.159	8	0.364	0.205	0.005
12	DRB1*08:01	43	0.045	3	0.136	0.092	0.022
13	DRB1*11:02	7	0.007	1	0.045	0.038	0.024

**FIGURE 3:**
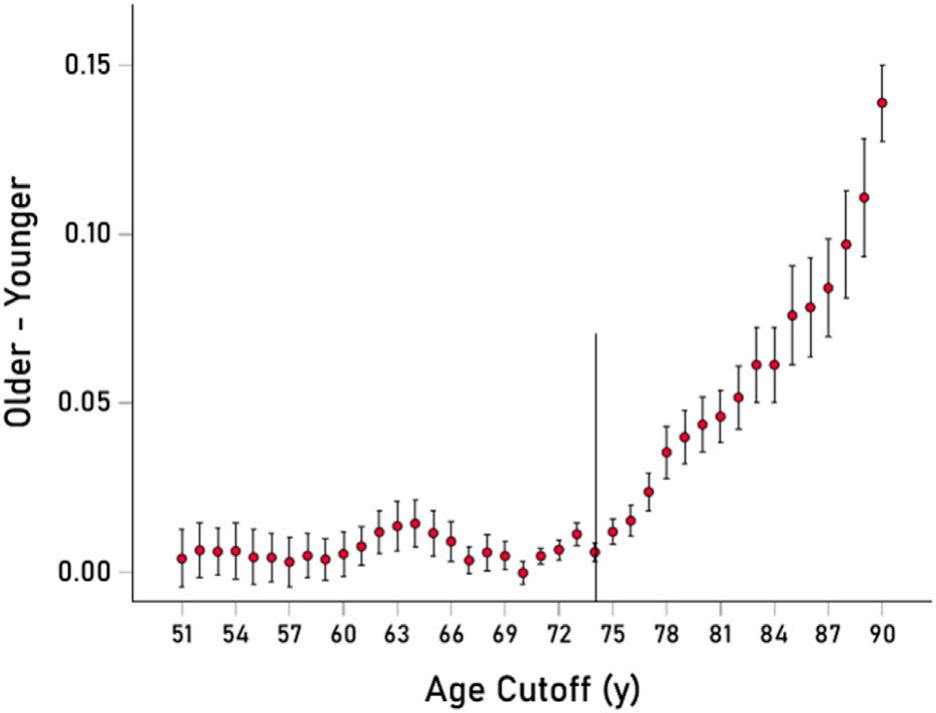
The mean ± SEM of the difference of proportions of the 13 VOAs (older–younger group samples) is plotted against the age cut-off threshold for younger/older group definitions. Thin vertical line is at 74 y. See text for details.

In a different analysis, we computed proportions of occurrence of the 13 VOAs across age starting at 51 years and moving forward every year–i.e., those younger than 51 vs those 51 years and older, those younger than 52 vs those 52 years and older, and so forth. The time course of the difference of proportions between the older and younger groups is shown in Figure 3. It can be seen that a systematic, monotonic increase in proportions (“enrichment”) of the 13 VOAs starts at age 74, marked by a thin vertical line in Figure 3.

### 3.3 Association of virus PBA with longevity

**FIGURE 4:**
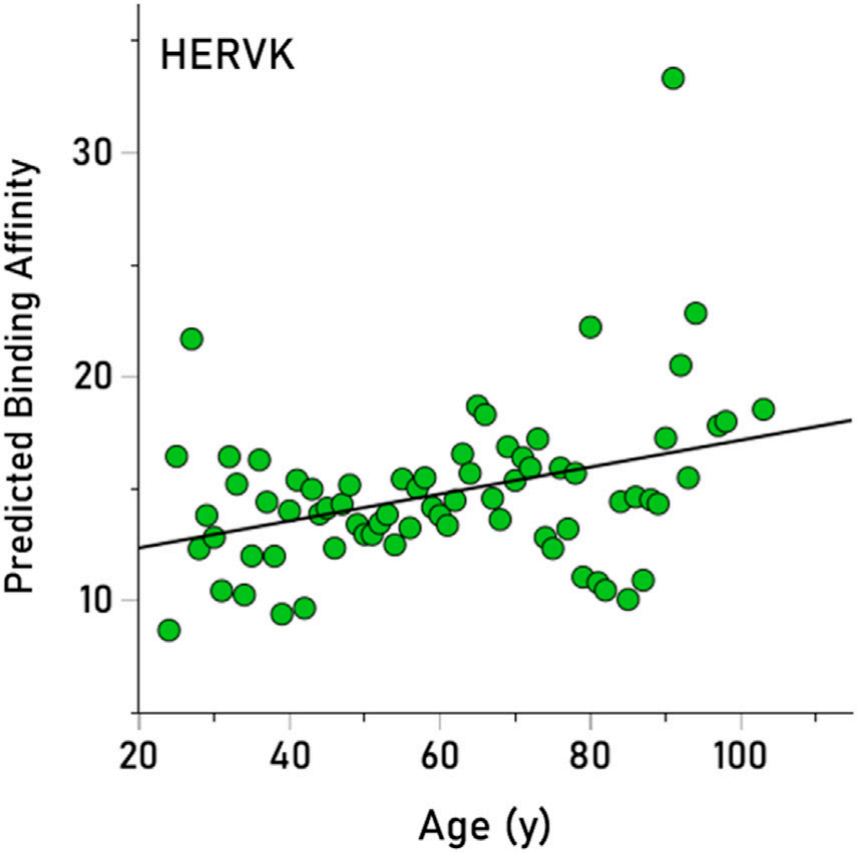
The average annual median PBA of HERVK is plotted against the year. See text for details.

Here we tested the hypothesis that longevity could be associated with higher PBA for viruses that impede it, given that higher PBA entails a higher chance in eliminating a harmful virus and, hence, a higher chance of living longer. We obtained PBA estimates for 235/244 (96.3%) alleles, since 9 alleles could not be analyzed by the binding affinity tool (These alleles occurred in 10 participants, hence the number of participants carrying, as a pool, 235 alleles was 976.) We tested the hypothesis above as follows: (i) Given that there are 12 HLA alleles per participant (6 of HLA-I and 6 of HLA-II classical genes, namely, A, B, C for HLA-I and DPB1, DQB1, DRB1 for HLA-II) and 13 virus PBAs per allele, we first obtained the median PBA for each participant and virus, from the set of these12 alleles. This yielded a matrix of 976 participants x 13 median virus PBAs. (ii) Next, we performed a stepwise multiple linear regression where the age of the participant was the dependent variable and the 13 median virus PBAs were the independent variables. This analysis yielded only one statistically significant effect, namely, a positive association between age and HERVK (P = 0.005). (iii) This effect was further quantified and visualized using a simple linear regression between the annual averages of age and HERVK median PBA (across all participants). The result is plotted in Figure 4, illustrating the finding that HERVK PBA increases significantly with age (r = 0.357, P = 0.002) (It can be seen in Figure 4 that there is a high PBA value; if that is removed from the analysis, the positive association between and HERVK PBA remains strong and highly significant [r = 0.326, P = 0.005], attesting to the robustness of this effect).

### 3.4 HLA alleles in the very old and their association with virus PBA

**FIGURE 5:**
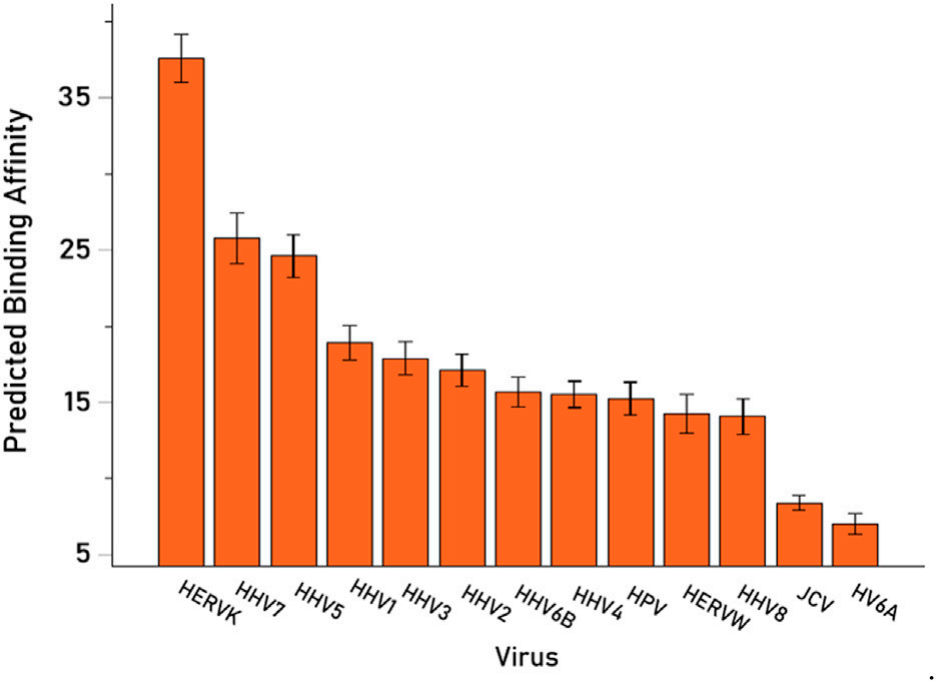
Mean (±95% Confidence Intervals) PBA of the 13 viruses studied for the 11 VOA alleles (alleles C*02:02 and C*03:02 could not by modeled by the IEDB NetMHCpan affinity tool).

**FIGURE 6:**
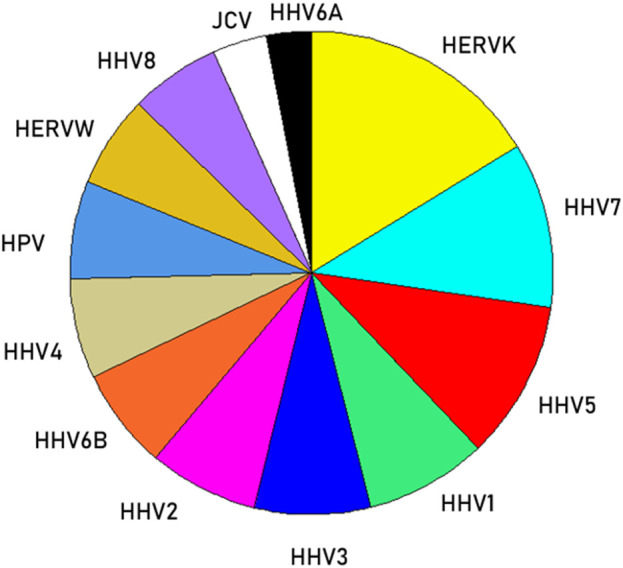
Pie plot to illustrate the relative (%) contribution of viral PBAs to VOAs (Table 5).

**FIGURE 7:**
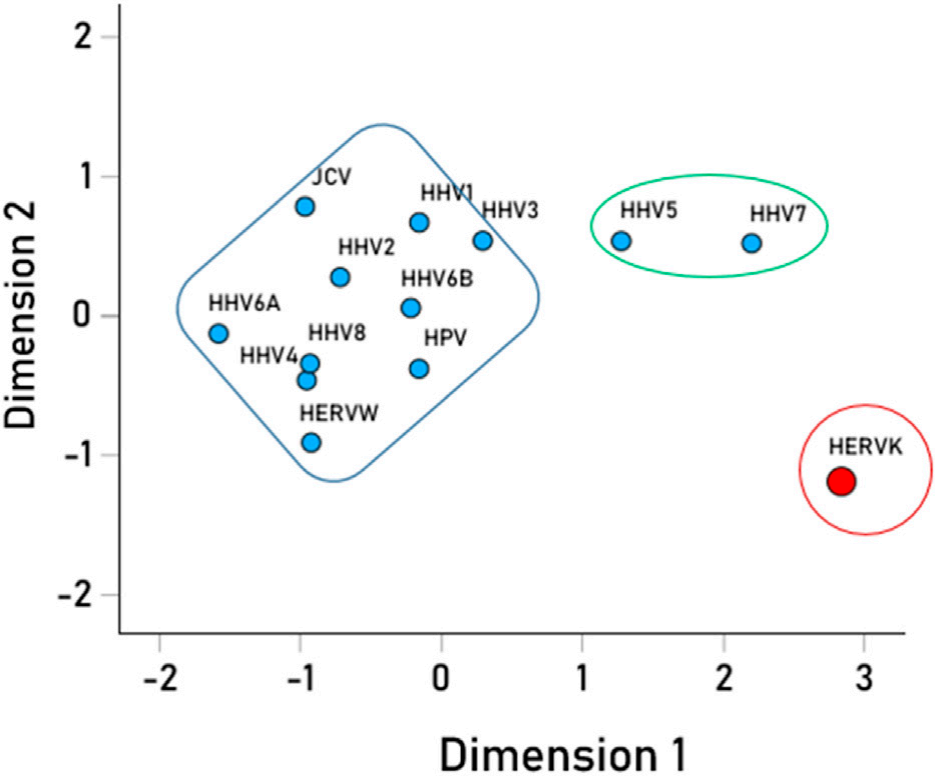
Multidimensional scaling plot of the viral PBAs. The clusters outlined were the outcomes of the K-means clustering analysis of the PBA X-Y coordinates.

We then analyzed the PBAs of the 13 viruses in 11/13 VOA alleles (alleles C*02:02 and C*03:02 could not be modeled by the IEDB NetMHCpan affinity tool) using a repeated-measures analysis of variance (ANOVA), where virus PBAs were the dependent variables. We found that the PBA for HERVK was significantly higher than those of all the other viruses (P < 0.001, Bonferroni corrected for multiple comparisons) (Figure 5). The relative contribution of specific viral PBAs of the 11 VOAs, considered as an aggregate, is given in Table 5 (as percent of the sum of all 13 PBAs) and illustrated in Figure 6. It can be seen that HERVK PBA contributed the most (16.2%), followed by HHV7 (11.11%) and HHV5 (10.61%); collectively, the PBAs of these 3 viruses accounted for 37.92% of the total PBA of the 11 VOA alleles. Finally, the grouping of viral PBAs was evaluated using MDS and is shown in Figure 7. It can be seen that HERVK is farthest away from all other viruses, and that HHV7 and HHV5 are well separated from the remaining viruses. The apparent 3 cluster formation in the MDS plot of Figure 7 was documented by the result of a K-means clustering analysis of the 13 X-Y viral MDS coordinates, which assigned each viral PBA to one of the 3 distinct clusters, as demarcated in Figure 7. The cluster separation was further evaluated by performing a multivariate analysis of variance (MANOVA), where the MDS X and Y coordinates were the dependent variables and the cluster assignment was a fixed factor. This analysis yielded a highly statistically significant cluster separation (Hoteling’s Trace test = 6.019, P = 0.0001).

**TABLE 5:** Relative (%) contributions of the 13 viral PBAs to the VOAs. See text for details.

Rank	Virus	% PBA	Cumulative %
1	HERVK	16.20	16.20
2	HHV7	11.11	27.31
3	HHV5	10.61	37.92
4	HHV1	8.15	46.07
5	HHV3	7.71	53.78
6	HHV2	7.37	61.15
7	HHV6B	6.75	67.90
8	HHV4	6.69	74.59
9	HPV	6.57	81.16
10	HERVW	6.14	87.30
11	HHV8	6.06	93.36
12	JCV	3.62	96.98
13	HHV6A	3.02	100.00
	Total	100.00	

### 3.5 ApoE

ApoE genotype is shown in Table 6. ApoE data were missing for three of the individuals in the younger group. The ApoE genotype distribution did not differ between the two groups (P = 0.898, chi-square test). There was also no difference in the proportion of the E2 allele (E2/E2, E2/E3) and E4 allele (E3/E4, E4/E4) between the two groups (E2: P = 0.978; E4: P = 0.379; Wilson test of two proportions).

**TABLE 6:** ApoE composition of very old and younger groups.

	Age group		
ApoE	<90 years	≥90 years	Total
22	6	0	6
	0.60%	0.00%	0.60%
23	127	3	130
	13.20%	13.60%	13.20%
24	23	1	24
	2.40%	4.50%	2.40%
33	550	14	564
	57.20%	63.60%	57.40%
34	226	4	230
	23.50%	18.20%	23.40%
44	29	0	29
	3.00%	0.00%	3.00%
Total	961	22	983
	100.00%	100.00%	100.00%

## 4 Discussion

Here we evaluated longevity with respect to HLA and tested the hypothesis that longevity may be related to the enhanced ability to counter common viruses that have been implicated in morbidity and mortality. We first identified 244 HLA alleles in this sample and documented a highly significant positive association between the predicted binding affinity of those alleles to HERVK (but not any of the other viruses) and age. We then identified 13 HLA alleles (out of 244) that were more common among very old individuals than their younger counterparts, suggesting that those alleles may promote longevity. We documented that the set of very old alleles was associated with significantly higher binding affinity to HERVK, followed by HHV5 and HHV7, than to other common viruses. Taken together, the present findings suggest that specific HLA alleles may promote longevity via enhanced ability to mount immune responses to HERVK, an endogenous retrovirus that may otherwise contribute to morbidity and mortality.

### 4.1 Methodological considerations

With respect to HLA genotyping, we analyzed alleles at 2-field resolution, where the first field defines the allele group that corresponds to the serologically defined specificity of the HLA protein and the second field indicates differences in the DNA sequence that lead to a difference in the amino acid sequence of the resulting protein. The third field indicates synonymous DNA substitutions in the coding region, whereas the fourth field refers to differences in the non-coding regions. Since differences in the third and fourth fields do not have any influence on the resulting protein, which was the focus of this study, we did not use them in this analysis. However, ambiguities stemming from match/mismatches in the coding (third field) and/or non-coding (fourth field) DNA sequences (Voorter et al., 2014) are likely to be important with regard to successful transplantation (Mayor et al., 2021).

Finally, with respect to epitope length, although various lengths are possible, it is recommended that 9-mers for HLA-I (Lafuente and Reche, 2009) and 15-mers for HLA-II) (Rudensky et al., 1991) are most suitable. More specifically, for HLA-I, “… most MHCI-peptide ligands have nine residues (they are 9-mers), making models for the prediction of 9-mer binders preferable” (23, page 3211), as exemplified by the choice to use 9-mers in a detailed biophysical study of antigen presentation to HLA-I molecules (Garstka et al., 2015).

### 4.2 Viruses and disease associations

HERVK is part of a broad class of human endogenous retroviruses that are integrated into the human genome. Among the endogenous retroviruses, HERVK (particularly the HML-2 subgroup evaluated here) is the most transcriptionally active, possessing open reading frames allowing for coding of proteins (Garcia-Montojo et al., 2018) that are found in several tissues (Flockerzi et al., 2008). HERVK is minimally expressed in healthy adult cells but is upregulated and expressed, producing virus-like particles, in various conditions (Shin et al., 2023) and has been associated with cancers (Rivas et al., 2022; Curty et al., 2020), neurological conditions, diabetes, and autoimmune disorders (Garcia-Montojo et al., 2018; Xue et al., 2020). HERVK has also been associated with cellular senescence and tissue aging (Liu et al., 2023). Here, the predicted binding affinity of HERVK was highly significantly and positively associated with age. In addition, the 13 alleles identified in the very old group were shown to confer enhanced protection against HERVK, suggesting reduced HERVK-associated risks in the very old group. Notably, the 13 very old alleles were also significantly associated with the predicted binding affinities of HHV7 and HHV5. Several studies, reviewed elsewhere (Chen et al., 2019) have documented that infection with herpes viruses, among others, can induce HERV transactivation, contributing to virus-associated diseases and tumors. Thus, possessing the very old alleles identified here may promote longevity by facilitating not only an immune response to HERVK but also to HHV7 and HHV5 thereby reducing herpes virus-related HERV transactivation and long-term impacts on HERVK-associated morbidity and mortality. It should be noted that these 13 alleles were not absent in the younger group but were significantly less frequent; thus, members of the younger cohort who possess these 13 alleles would presumably be afforded greater longevity.

### 4.3 HLA and longevity

Previous research has documented an association between the HLA-DRB1 region and longevity (Joshi et al., 2017). Similarly, we found that 3 of the 13 alleles which were more frequent in the very old group belong to the Class II DRB1 gene. Here, however, several other HLA genes including Class I HLA-A (3 alleles), HLA-B (3 alleles), HLA-C (3 alleles), and Class II DQB1 (1 allele) were also more frequent in the very old group. Thus, the findings of our study which was based on high-resolution genotyping (vs imputation), confirm those of previous GWAS studies and document the relevance of additional HLA genes to longevity. Indeed, several of the very old alleles have been associated with disease protection. For instance, HLA-A*02:05 and HLA-A*03:01 are protective against severe clinical manifestations of SARS-CoV-2 (Littera et al., 2020; Shkurnikov et al., 2021), DRB1*01:01 is protective against MS (Mamedov et al., 2020), and DRB1*11:02 protects against rheumatoid arthritis (Cruz-Tapias et al., 2013). Perhaps the most well-investigated is HLA-B*27:05 which has been associated with risk for ankylosing spondylitis (Khan, 2023), yet superior immune control of HIV (International et al., 2010) and hepatitis C virus (Neumann-Haefelin et al., 2010). Thus, this group of alleles that are enhanced in very old individuals have been shown to protect against certain viruses and diseases, presumably promoting longevity.

### 4.4 Host-virus interactions

When considering protective or susceptibility effects related to HLA, it is crucial to keep in mind the role of HLA in host protection via signaling the immune system to eliminate foreign antigens. That is, protective or susceptibility effects are not conferred by HLA alone but by interactions between an individual’s HLA composition and exposure to viruses and other foreign antigens. Consequently, a given allele may preferentially bind and mount an immune response to certain pathogens over others. Indeed, this is what was documented for the 13 very old alleles here with regard to the 13 viruses we investigated. That is, as a group of alleles, those that were more common in the very old group had enhanced ability to eliminate HERVK relative to others.

### 4.5 ApoE and longevity

Notably, apolipoprotein E composition did not differ between the very old cohort and the younger cohort in the present study. Some previous research has found that the E2 allele is associated with increased longevity and the E4 allele with decreased longevity (Sebastiani et al., 2019), although the overall findings with regard to apoE and longevity are mixed (Abondio et al., 2019). ApoE is most prominently implicated in dementia (Serrano-Pozo et al., 2021); individuals who had been diagnosed with dementia were not included in the present study.

### 4.6 Conclusions and limitations

In summary, the findings of the present study suggest that longevity is associated with enhanced immune response to specific common viruses–particularly, HERVK–conferred by specific HLA alleles. These novel findings, which highlight the effect of interaction between host immunogenetics and virus exposure on longevity, need to be considered in light of several qualifications. First, the findings here are based on *in silico* analyses; future studies evaluating HLA and HERVK antigen-specific CD8 T cell receptor repertoire and in long-living individuals are warranted to further substantiate the conclusions herein. Second, we focused exclusively on the 244 alleles that were documented in our sample and particularly on 13 alleles that were enriched in the very old group of participants. That does not preclude the possibility of other alleles also mounting robust immune responses to HERVK. Indeed, in light of the role of HLA in host protection against viruses, it is likely that additional alleles that were equally present (or absent) in both groups bind with high affinity to the common viruses investigated here. In addition, it is worth noting that the current findings are specific to longevity and may not equate with overall health as only health conditions that would interfere with comprehension or participation were exclusionary. Finally, although the viruses and HLA alleles are relatively common globally, the frequencies of both are known to vary geographically (Looker et al., 2017; Adane and Getawa, 2021; Prugnolle et al., 2005; Singh et al., 2007). Although additional studies are warranted to determine whether the longevity alleles identified here generalize to other populations, all of the 13 alleles that were enriched in the very old group of participants here are common worldwide (Hurley et al., 2020), suggesting these alleles with high affinity to HERVK may promote global longevity.

## Data Availability

The raw data supporting the conclusions of this article will be made available by the authors, without undue reservation.
