# Supplementary material for: Immunogenetics of longevity and its association with human endogenous retrovirus K
Source: Front Aging. 2025 Feb 4;6:1471202. doi: 10.3389/fragi.2025.1471202 (PMC11832543; doi:10.3389/fragi.2025.1471202)
Supplement: Supplementary file 1 [file DataSheet1.docx]

**APPENDIX**

Amino acid sequences of the 9 HHV strains (Table 1), human polyoma virus (JCV), human papilloma virus, and human endogenous retroviruses HERVK and HERVW. Strain labels are from Uniprot (https://www.uniprot.org/uniprotkb/)

| HHV1: Q69091 · GD_HHV11 | Envelope glycoprotein D | 394 AA |
| --- | --- | --- |

MGGAAARLGAVILFVVIVGLHGVRSKYALVDASLKMADPNRFRGKDLPVLDQLTDPPGVRRVYHIQAGLPDPFQPPSLPITVYYAVLERACRSVLLNAPSEAPQIVRGASEDVRKQPYNLTIAWFRMGGNCAIPITVMEYTECSYNKSLGACPIRTQPRWNYYDSFSAVSEDNLGFLMHAPAFETAGTYLRLVKINDWTEITQFILEHRAKGSCKYALPLRIPPSACLSPQAYQQGVTVDSIGMLPRFIPENQRTVAVYSLKIAGWHGPKAPYTSTLLPPELSETPNATQPELAPEDPEDSALLEDPVGTVAPQIPPNWHIPSIQDAATPYHPPATPNNMGLIAGAVGGSLLAALVICGIVYWMRRHTQKAPKRIRLPHIREDDQPSSHQPLFY

| HHV2: P03172 · GD_HHV23 | Envelope glycoprotein D | 393 AA |
| --- | --- | --- |

MGRLTSGVGTAALLVVAVGLRVVCAKYALADPSLKMADPNRFRGKNLPVLDRLTDPPGVKRVYHIQPSLEDPFQPPSIPITVYYAVLERACRSVLLHAPSEAPQIVRGASDEARKHTYNLTIAWYRMGDNCAIPITVMEYTECPYNKSLGVCPIRTQPRWSYYDSFSAVSEDNLGFLMHAPAFETAGTYLRLVKINDWTEITQFILEHRARASCKYALPLRIPPAACLTSKAYQQGVTVDSIGMLPRFIPENQRTVALYSLKIAGWHGPKPPYTSTLLPPELSDTTNATQPELVPEDPEDSALLEDPAGTVSSQIPPNWHIPSIQDVAPHHAPAAPSNPGLIIGALAGSTLAVLVIGGIAFWVRRRAQMAPKRLRLPHIRDDDAPPSHQPLFY

| HHV3: Q9J3M8 · GE_VZVO | Envelope glycoprotein E | 623 AA |
| --- | --- | --- |

MGTVNKPVVGVLMGFGIITGTLRITNPVRASVLRYDDFHIDEDKLDTNSVYEPYYHSDHAESSWVNRGESSRKAYDHNSPYIWPRNDYDGFLENAHEHHGVYNQGRGIDSGERLMQPTQMSAQEDLGDDTGIHVIPTLNGDDRHKIVNVDQRQYGDVFKGDLNPKPQGQRLIEVSVEENHPFTLRAPIQRIYGVRYTETWSFLPSLTCTGDAAPAIQHICLKHTTCFQDVVVDVDCAENTKEDQLAEISYRFQGKKEADQPWIVVNTSTLFDELELDPPEIEPGVLKVLRTEKQYLGVYIWNMRGSDGTSTYATFLVTWKGDEKTRNPTPAVTPQPRGAEFHMWNYHSHVFSVGDTFSLAMHLQYKIHEAPFDLLLEWLYVPIDPTCQPMRLYSTCLYHPNAPQCLSHMNSGCTFTSPHLAQRVASTVYQNCEHADNYTAYCLGISHMEPSFGLILHDGGTTLKFVDTPESLSGLYVFVVYFNGHVEAVAYTVVSTVDHFVNAIEERGFPPTAGQPPATTKPKEITPVNPGTSPLLRYAAWTGGLAAVVLLCLVIFLICTAKRMRVKAYRVDKSPYNQSMYYAGLPVDDFEDSESTDTEEEFGNAIGGSHGGSSYTVYIDKTR

| HHV4: P03188 · GB_EBVB9 | Envelope glycoprotein B | 857 AA |
| --- | --- | --- |

MTRRRVLSVVVLLAALACRLGAQTPEQPAPPATTVQPTATRQQTSFPFRVCELSSHGDLFRFSSDIQCPSFGTRENHTEGLLMVFKDNIIPYSFKVRSYTKIVTNILIYNGWYADSVTNRHEEKFSVDSYETDQMDTIYQCYNAVKMTKDGLTRVYVDRDGVNITVNLKPTGGLANGVRRYASQTELYDAPGWLIWTYRTRTTVNCLITDMMAKSNSPFDFFVTTTGQTVEMSPFYDGKNKETFHERADSFHVRTNYKIVDYDNRGTNPQGERRAFLDKGTYTLSWKLENRTAYCPLQHWQTFDSTIATETGKSIHFVTDEGTSSFVTNTTVGIELPDAFKCIEEQVNKTMHEKYEAVQDRYTKGQEAITYFITSGGLLLAWLPLTPRSLATVKNLTELTTPTSSPPSSPSPPAPSAARGSTPAAVLRRRRRDAGNATTPVPPTAPGKSLGTLNNPATVQIQFAYDSLRRQINRMLGDLARAWCLEQKRQNMVLRELTKINPTTVMSSIYGKAVAAKRLGDVISVSQCVPVNQATVTLRKSMRVPGSETMCYSRPLVSFSFINDTKTYEGQLGTDNEIFLTKKMTEVCQATSQYYFQSGNEIHVYNDYHHFKTIELDGIATLQTFISLNTSLIENIDFASLELYSRDEQRASNVFDLEGIFREYNFQAQNIAGLRKDLDNAVSNGRNQFVDGLGELMDSLGSVGQSITNLVSTVGGLFSSLVSGFISFFKNPFGGMLILVLVAGVVILVISLTRRTRQMSQQPVQMLYPGIDELAQQHASGEGPGINPISKTELQAIMLALHEQNQEQKRAAQRAAGPSVASRALQAARDRFPGLRRRRYHDPETAAALLGEAETEF

| HHV5: P06473 · GB_HCMVA | Envelope glycoprotein B | gB | 906 AA |
| --- | --- | --- | --- |

MESRIWCLVVCVNLCIVCLGAAVSSSSTSHATSSTHNGSHTSRTTSAQTRSVYSQHVTSSEAVSHRANETIYNTTLKYGDVVGVNTTKYPYRVCSMAQGTDLIRFERNIICTSMKPINEDLDEGIMVVYKRNIVAHTFKVRVYQKVLTFRRSYAYIYTTYLLGSNTEYVAPPMWEIHHINKFAQCYSSYSRVIGGTVFVAYHRDSYENKTMQLIPDDYSNTHSTRYVTVKDQWHSRGSTWLYRETCNLNCMLTITTARSKYPYHFFATSTGDVVYISPFYNGTNRNASYFGENADKFFIFPNYTIVSDFGRPNAAPETHRLVAFLERADSVISWDIQDEKNVTCQLTFWEASERTIRSEAEDSYHFSSAKMTATFLSKKQEVNMSDSALDCVRDEAINKLQQIFNTSYNQTYEKYGNVSVFETSGGLVVFWQGIKQKSLVELERLANRSSLNITHRTRRSTSDNNTTHLSSMESVHNLVYAQLQFTYDTLRGYINRALAQIAEAWCVDQRRTLEVFKELSKINPSAILSAIYNKPIAARFMGDVLGLASCVTINQTSVKVLRDMNVKESPGRCYSRPVVIFNFANSSYVQYGQLGEDNEILLGNHRTEECQLPSLKIFIAGNSAYEYVDYLFKRMIDLSSISTVDSMIALDIDPLENTDFRVLELYSQKELRSSNVFDLEEIMREFNSYKQRVKYVEDKVVDPLPPYLKGLDDLMSGLGAAGKAVGVAIGAVGGAVASVVEGVATFLKNPFGAFTIILVAIAVVIITYLIYTRQRRLCTQPLQNLFPYLVSADGTTVTSGSTKDTSLQAPPSYEESVYNSGRKGPGPPSSDASTAAPPYTNEQAYQMLLALARLDAEQRAQQNGTDSLDGQTGTQDKGQKPNLLDRLRHRKNGYRHLKDSDEEENV

| HHV6A: P0DOE0 · GQ2_HHV6U | Envelope glycoprotein Q2 | 214 AA |
| --- | --- | --- |

MHFLVVYILIHFHAYRGMAALPLFSTLPKITSCCDSYVVINSSTSVSSLISTCLDGEILFQNEGQKFCRPLTDNRTIVYTMQDQVQKPLSVTWMDFNLVISDYGRDVINNLTKSAMLARKNGPRYLQMENGPRYLQMETRISDLFRHECYQDNYYVLDKKLQMFYPTTHSNELLFYPSEATLPSPWQEPPFSSPWPEPTFPSRWYWLLLNYTNY

| HHV6B: Q9QJ11 · GQ1_HHV6Z | Envelope glycoprotein Q1 | 516 AA |
| --- | --- | --- |

MRPPRRSAPILVCAISMATALSNATVHRDAGTVESTPPPDDEDNYTAKYYDDSIYFNIYDGTNPTPRRRTLPEIISKFSTSEMSRLGGLKAFVPVDYTPTTTLEDIEDLLNYAICDDNSCGCLIETEARXMFGDIIICVPLSAESRGVRNLKSRIMPMGLSQILSSGLGLHFSLLYGAFGSNYNSLAYMERLKPLTAMTAIAFCPMTSKLELRQNYRLEKARXNLIVNIELLKIQNHGGQTIKTLTSFAIVRKDSDGQDWETCTRFASVSIEDILRSKPAANGTCCPPRDVHHDRPTLQSSNSWTRTEYFEPWQDVVDAYVPINDNHCPNDSYVVFQTLQGHEWCSRLNKNDTKNYLSSVLAFKNALYETEELMETIGMRLASQILSLVGQRGTSIRNIDPAIVSALWHSLPEKLTTTNIKYDIASPTHMSPALXTIFIQTGTSKQRFRNAGLLMVNNIFTVQARYSKQNMFEKKIYGYEHLGQALCEGGHVFYNPRDVYFQNIKMAATEPTVVRT

| HHV7: P52353 · GH_HHV7J | Envelope glycoprotein H | 690 AA |
| --- | --- | --- |

MYFYINSLLLIVSINGWKHWNILNSSICVNEKTNQTIIQPGLITFNFHDYNETRVYQIPKCLFGYTFVSNLFDSVNFDESFDQYKHRITRFFNPSTEKAVKIYAQKFQTNIKPVSHTKTITVSFLPLFYEKDVYFANVSEIRKLYYNQYICTLSNGLTDYLFPITERCVMRHYNYLNTVFMLALTPSFFIISVETGMDDVVFIFGNVSRIFFKAPFRKSSFIYRQTVSDDLLLITKKTTIERFYPFLKIDFLDDIWKQNYDISFLIAKFNKLATVYIMEGFCGKPVNKDTFHLMFLFGLTHFLYSTRGDGLLPLLEILNTHQSIITMGRFLEKCFKMTKSHLLYPEMEKLQNFQLVDYSYITSDLTIPISAKLAFLSLADGRIVTVPQNKWKEIENNIETLYEKHKLFTNLTQPERANLFLLSEIGNSLVFQEKIKRKIHVLLASLCNPLEMYFWTHMLDNVMDIETMFSPCATATRKDLTQRVVNNILSYKNLDAYTNKVMNTLSVYRKKRLDMFKSISCVSNEQAAFLTLPNITYTISSKYILAGTSFSVTSTVISTTIIITVVPLNSTCTPTNYKYSVKNIKPIYNISSHDCVFCESLVVEYDDIDGIIQFVYIMDDKQLLKLIDPDTNFIDVNPRTHYLLFLRNGSVFEITALDLKSSQVSIMLVLLYLIIIIIVLFGIYHVFRLF

| HHV8: F5HAK9 · GH_HHV8P | Envelope glycoprotein H | 730 AA |
| --- | --- | --- |

MQGLAFLAALACWRCISLTCGATGALPTTATTITRSATQLINGRTNLSIELEFNGTSFFLNWQNLLNVITEPALTELWTSAEVAEDLRVTLKKRQSLFFPNKTVVISGDGHRYTCEVPTSSQTYNITKGFNYSALPGHLGGFGINARLVLGDIFASKWSLFARDTPEYRVFYPMNVMAVKFSISIGNNESGVALYGVVSEDFVVVTLHNRSKEANETASHLLFGLPDSLPSLKGHATYDELTFARNAKYALVAILPKDSYQTLLTENYTRIFLNMTESTPLEFTRTIQTRIVSIEARRACAAQEAAPDIFLVLFQMLVAHFLVARGIAEHRFVEVDCVCRQYAELYFLRRISRLCMPTFTTVGYNHTTLGAVAATQIARVSATKLASLPRSSQETVLAMVQLGARDGAVPSSILEGIAMVVEHMYTAYTYVYTLGDTERKLMLDIHTVLTDSCPPKDSGVSEKLLRTYLMFTSMCTNIELGEMIARFSKPDSLNIYRAFSPCFLGLRYDLHPAKLRAEAPQSSALTRTAVARGTSGFAELLHALHLDSLNLIPAINCSKITADKIIATVPLPHVTYIISSEALSNAVVYEVSEIFLKSAMFISAIKPDCSGFNFSQIDRHIPIVYNISTPRRGCPLCDSVIMSYDESDGLQSLMYVTNERVQTNLFLDKSPFFDNNNLHIHYLWLRDNGTVVEIRGMYRRRAASALFLILSFIGFSGVIYFLYRLFSILY

| JC polyomavirus (JCV)  P03089 VP1_POVJC | Major capsid protein VP1 | 354 AA |
| --- | --- | --- |

MAPTKRKGERKDPVQVPKLLIRGGVEVLEVKTGVDSITEVECFLTPEMGDPDEHLRGFSKSISISDTFESDSPNRDMLPCYSVARIPLPNLNEDLTCGNILMWEAVTLKTEVIGVTSLMNVHSNGQATHDNGAGKPVQGTSFHFFSVGGEALELQGVLFNYRTKYPDGTIFPKNATVQSQVMNTEHKAYLDKNKAYPVECWVPDPTRNENTRYFGTLTGGENVPPVLHITNTATTVLLDEFGVGPLCKGDNLYLSAVDVCGMFTNRSGSQQWRGLSRYFKVQLRKRRVKNPYPISFLLTDLINRRTPRVDGQPMYGMDAQVEEVRVFEGTEELPGDPDMMRYVDKYGQLQTKML

| Human papillomavirus Q81007 | Major capsid protein L1 | 494 AA |
| --- | --- | --- |

TVYLPPVPVSKVVSTDEYVARTNIYYHAGTSRLLAVGHPYFPIKKPNNNKILVPKVSGLQYRVFRIHLPDPNKFGFPDTSFYNPDTQRLVWACVGVEVGRGQPLGVGISGHPLLNKLDDTENASAYAANAGVDNRECISMDYKQTQLCLIGCKPPIGEHWGKGSPCTNVAVNPGDCPPLELINTVIQDGDMVDTGFGAMDFTTLQANKSEVPLDICTSICKYPDYIKMVSEPYGDSLFFYLRREQMFVRHLFNRAGTVGENVPDDLYIKGSGSTANLASSNYFPTPSGSMVTSDAQIFNKPYWLQRAQGHNNGICWGNQLFVTVVDTTRSTNMSLCAAISTSETTYKNTNFKEYLRHGEEYDLQFIFQLCKITLTADVMTYIHSMNSTILEDWNFGLQPPPGGTLEDTYRFVTSQAIACQKHTPPAPKEDPLKKYTFWEVNLKEKFSADLDQFPLGRKFLLQAGLKAKPKFTLGKRKATPTTSSTSTTAKRKKR

| Human Endogenous Retrovirus HERV W Q9UQF0 | Envelope protein | 538 AA |
| --- | --- | --- |

MALPYHIFLFTVLLPSFTLTAPPPCRCMTSSSPYQEFLWRMQRPGNIDAPSYRSLSKGTPTFTAHTHMPRNCYHSATLCMHANTHYWTGKMINPSCPGGLGVTVCWTYFTQTGMSDGGGVQDQAREKHVKEVISQLTRVHGTSSPYKGLDLSKLHETLRTHTRLVSLFNTTLTGLHEVSAQNPTNCWICLPLNFRPYVSIPVPEQWNNFSTEINTTSVLVGPLVSNLEITHTSNLTCVKFSNTTYTTNSQCIRWVTPPTQIVCLPSGIFFVCGTSAYRCLNGSSESMCFLSFLVPPMTIYTEQDLYSYVISKPRNKRVPILPFVIGAGVLGALGTGIGGITTSTQFYYKLSQELNGDMERVADSLVTLQDQLNSLAAVVLQNRRALDLLTAERGGTCLFLGEECCYYVNQSGIVTEKVKEIRDRIQRRAEELRNTGPWGLLSQWMPWILPFLGPLAAIILLLLFGPCIFNLLVNFVSSRIEAVKLQMEPKMQSKTKIYRRPLDRPASPRSDVNDIKGTPPEEISAAQPLLRPNSAGSS

| Human endogenous retrovirus group K P10266 | 10 Pol protein | 1014 AA |
| --- | --- | --- |

NKSRKRRNRVSFLGAVTVEPPKPIPLTWKTEKPVWVNQWPLPKQKLEALHLLANEQLEKGHIEPSFSPWNSPVFVIQKKSGKWHTLTDLRAVNAVIQPMGPLQPGLPSPAMIPKDWPLIIIDLKDCFFTIPLAEQDCEKFAFTIPAINNKEPATRFQWKVLPQGMLNSPTICQTFVGRALQPVREKFSDCYIIHYIDDILCAAETKDKLIDCYTFLQAEVANAGLAIASDKIQTSTPFHYLGMQIENRKIKPQKIEIRKDTLKTLNDFQKLLGDINWIRPTLGIPTYAMSNLFSILRGDSDLNSQRILTPEATKEIKLVEEKIQSAQINRIDPLAPLQLLIFATAHSPTGIIIQNTDLVEWSFLPHSTVKTFTLYLDQIATLIGQTRLRITKLCGNDPDKIVVPLTKEQVRQAFINSGAWQIGLANFVGLIDNHYPKTKIFQFLKLTTWILPKITRREPLENALTVFTDGSSNGKAAYTGPKERVIKTPYQSAQRDELVAVITVLQDFDQPINIISDSAYVVQATRDVETALIKYSMDDQLNQLFNLLQQTVRKRNFPFYITYIRAHTNLPGPLTKANEQADLLVSSALIKAQELHALTHVNAAGLKNKFDVTWKQAKDIVQHCTQCQVLHLPTQEAGVNPRGLCPNALWQMDVTHVPSFGRLSYVHVTVDTYSHFIWATCQTGESTSHVKKHLLSCFAVMGVPEKIKTDNGPGYCSKAFQKFLSQWKISHTTGIPYNSQGQAIVERTNRTLKTQLVKQKEGGDSKECTTPQMQLNLALYTLNFLNIYRNQTTTSAEQHLTGKKNSPHEGKLIWWKDNKNKTWEIGKVITWGRGFACVSPGENQLPVWLPTRHLKFYNEPIGDAKKRASTEMVTPVTWMDNPIEVYVNDSIWVPGPIDDRCPAKPEEEGMMINISIGYRYPPICLGRAPGCLMPAVQNWLVEVPTVSPISRFTYHMVSGMSLRPRVNYLQDFSYQRSLKFRPKGKPCPKEIPKESKNTEVLVWEECVANSAVIL
